# Intranasal boosting with RBD-HR protein vaccine elicits robust mucosal and systemic immune responses

**DOI:** 10.1016/j.gendis.2023.06.035

**Published:** 2023-08-03

**Authors:** Li Chen, Wenyan Ren, Hong Lei, Jiayu Wang, Haiying Que, Dandan Wan, Aqu Alu, Dandan Peng, Minyang Fu, Weiqi Hong, Yuhe Huang, Xiangrong Song, Guangwen Lu, Xiawei Wei

**Affiliations:** Laboratory of Aging Research and Cancer Drug Target, State Key Laboratory of Biotherapy and Cancer Center, National Clinical Research Center for Geriatrics, West China Hospital, Sichuan University, Chengdu, Sichuan 610041, China

**Keywords:** Heterologous immunization, Intranasal immunization, mRNA vaccine, SARS-CoV-2, Subunit protein vaccine

## Abstract

The emergence of severe acute respiratory syndrome coronavirus 2 (SARS-CoV-2) variants has decreased the efficacy of SARS-CoV-2 vaccines in containing coronavirus disease 2019 (COVID-19) over time, and booster vaccination strategies are urgently necessitated to achieve sufficient protection. Intranasal immunization can improve mucosal immunity, offering protection against the infection and sustaining the spread of SARS-CoV-2. In this study, an intranasal booster of the RBD-HR vaccine after two doses of the mRNA vaccine significantly increased the levels of specific binding antibodies in serum, nasal lavage fluid, and bronchoalveolar lavage fluid compared with only two doses of mRNA vaccine. After intranasal boosting with the RBD-HR vaccine, the levels of serum neutralizing antibodies against prototype and variant strains of SARS-CoV-2 pseudoviruses were markedly higher than those in mice receiving mRNA vaccine alone, and intranasal boosting with the RBD-HR vaccine also inhibited the binding of RBD to hACE2 receptors. Furthermore, the heterologous intranasal immunization regimen promoted extensive memory T cell responses and activated CD103^+^ dendritic cells in the respiratory mucosa, and potently enhanced the formation of T follicular helper cells and germinal center B cells in vital immune organs, including mediastinal lymph nodes, inguinal lymph nodes, and spleen. Collectively, these data infer that heterologous intranasal boosting with the RBD-HR vaccine elicited broad protective immunity against SARS-CoV-2 both locally and systemically.

## Introduction

The ongoing coronavirus disease 2019 (COVID-19) pandemic, caused by the continual emergence of severe acute respiratory syndrome coronavirus 2 (SARS-CoV-2) variants, remains a significant public health threat, with more than 759 million infections and 6.9 million deaths worldwide since the outbreak.^1^ Vaccination remains one of the most effective measures to trigger robust immune responses in individuals and to contain SARS-CoV-2 transmission through the respiratory tract. Indeed, tremendous efforts have been made to develop multiple SARS-CoV-2 vaccine candidates, chiefly including inactivated vaccines, protein subunit vaccines, viral vector vaccines, and nucleic acid vaccines based on various biological technologies.^2^ With the constant evolution of SARS-CoV-2, the efficacy of the vaccines containing COVID-19 wanes over time after the completion of the primary vaccine series.^3^ To overcome the limited immunogenicity of prime vaccination and meet the challenges posed by constant SARS-CoV-2 variants, a prime-boost vaccination schedule has become a necessary trend to establish herd immunity.^4^

Boosting with homologous and heterologous vaccines effectively induces antibody affinity maturation, cellular immune response, and sustained long-term immunogenicity against SARS-CoV-2. Numerous studies demonstrated that heterologous prime-boost strategies resulted in increases in the levels of specific binding antibodies and neutralizing antibodies compared with homologous booster doses. For instance, the combination of a heterologous prime-boost schedule with ChAdOx1 nCoV-19 (ChAd) and BNT162b2 (BNT) elicited higher levels of spike (S)-specific IgG antibodies, higher levels of neutralizing antibodies, and robust S-specific T cell responses compared with two homologous doses of ChAd.^5^^,^^6^ After intramuscularly priming with inactivated vaccine, heterologous vaccination with subunit vaccine, adenovirus-vectored vaccine, or mRNA vaccine enhanced both humoral and cellular immune responses compared with homologous immunization with inactivated vaccine.^7^ Additionally, intramuscular boosting with protein subunit vaccines after priming with inactivated vaccines significantly promoted the generation of anti-RBD antibodies and distinct cross-neutralizing activities against prototype and mutated SARS-CoV-2.^8^ Both systemic and mucosal immune responses are indispensable in restraining respiratory viral infections during the COVID-19 pandemic. Despite intramuscular injection with SARS-CoV-2 vaccines eliciting humoral and cellular immune responses, intramuscular vaccination fails to generate robust mucosal immunity to directly defend against infection and clear SARS-CoV-2 residing in the respiratory tract.

Given that SARS-CoV-2 primarily invades the body from the proximal to the distal respiratory tract, it is critical to enhance the protective immune response within respiratory mucosa. Intranasal vaccination is considered to be an effective approach for eliciting mucosal and systemic immune responses against SARS-CoV-2. According to an earlier study, intranasal delivery of two doses of an adjuvanted S protein-based vaccine evoked potent mucosal and systemic memory T cell responses, protecting K18-hACE2tg mice against the lethal SARS-CoV-2.^9^ Besides, intranasal administration of three doses of recombinant RBD vaccine adjuvanted with polyethyleneimine or aluminium oxyhydroxide gel induced considerably high titers of IgG antibodies, neutralizing antibodies, as well as antigen-specific T cell responses and strong mucosal immunity, including mucosal secretory IgA (SIgA) and lung-resident memory T cells (T_RM_).^10^, ^11^, ^12^ However, clinical data indicated that relying solely on intranasal vaccinations to protect humans from SARS-CoV-2 without pre-existing immunity may result in suboptimal immunogenicity, providing evidence that heterologous prime-boost vaccinations can induce robust immunity against the virus.^13^ While intramuscular immunization with mRNA vaccines alone elicited weak respiratory mucosal neutralizing antibody responses, an intranasal booster of S-trimer with a cGAMP or Ad5-S vaccine after intramuscular priming with two doses of an mRNA vaccine enhanced the production of S1- and RBD-specific IgA and neutralizing antibodies against D614G, Delta, and BA.1.1, as well as mucosal CD8^+^ and CD4^+^ T cell responses in bronchoalveolar lavage fluid (BALF).^14^ In addition, intramuscular priming with DNA vaccines or mRNA vaccines followed by intranasal administration with an adenovirus-vectored vaccine contributed to systemic and mucosal immune responses against SARS-CoV-2, consisting of high levels of IgG, IgG1, IgG2a, mucosal SIgA, and neutralizing antibodies in serum and BALF. Subsequent results demonstrated that S-specific T cell responses, circulating memory T cells, and resident memory T cells were efficiently generated via mucosal boosting with adenovirus-vectored vaccines.^15^

This study aimed to investigate the immunogenicity of intranasal boosting with the RBD-HR vaccine after prior systemic priming with two doses of mRNA vaccine in murine models. Our results revealed that intranasal boosting with RBD-HR vaccine elicited robust mucosal and systemic immune responses, indicating that intramuscular priming with mRNA vaccines plus intranasal boost with protein vaccines is a promising heterologous immunization strategy to control the COVID-19 pandemic.

## Materials and methods

### Cell culture

HEK293T cells were procured from the American Type Culture Collection and constructed by transduction of human angiotensin I converting enzyme 2 (hACE2) into HEK293T cells and subjected to stable cell selection to obtain 293T/hACE2 cells. HEK293T and 293T/hACE2 cells were cultivated at 37 °C in the Dulbecco's modified Eagle medium (C11885500BT, Thermo Fisher Scientific, USA) supplemented with 10% fetal bovine serum (st30-3302, PAN-Biotech, USA) and 1% penicillin-streptomycin (ST488, Beyotime, China).

### Immunization

**Figure 1:**
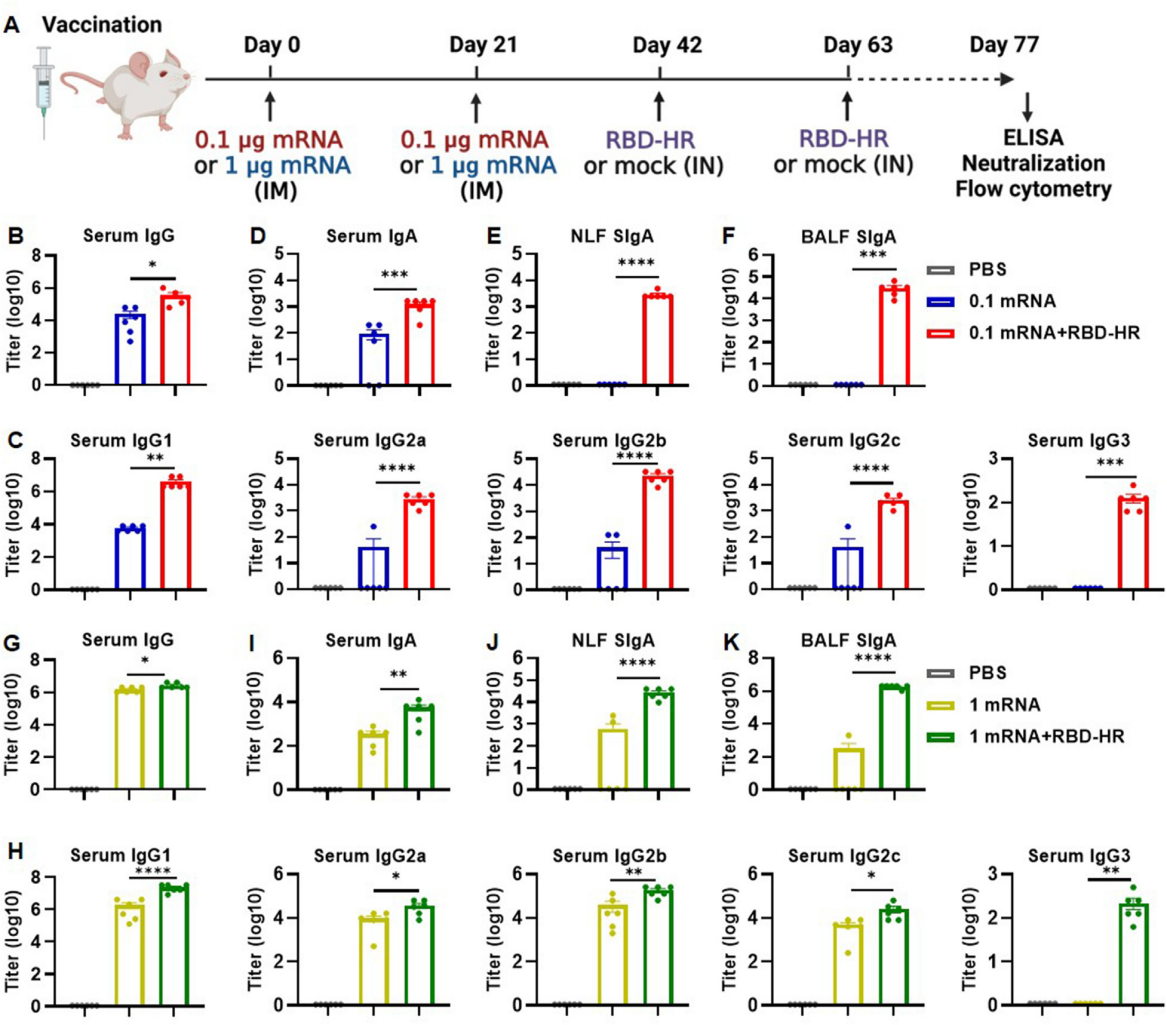
Intranasal boosting with RBD-HR protein vaccine elicited a robust humoral response. **(A)** A sequential vaccination schedule of two doses of mRNA vaccine followed by two doses of RBD-HR vaccine. This diagram was created with BioRender. **(B, G)** The levels of RBD-HR-specific IgG titer in the sera. **(D, I)** The levels of RBD-HR-specific IgA titer in the sera. **(E, J)** The levels of RBD-HR-specific SIgA titer in the NLF. **(F, K)** The levels of RBD-HR-specific SIgA titer in the BALF. **(C, H)** RBD-HR-specific IgG1, IgG2a, IgG2b, IgG2c, and IgG3 titers in the sera. Results were expressed as mean ± SEM, and *P* values were calculated by one-way ANOVA. *n* = 6; ^∗^*P* < 0.05, ^∗∗^*P* < 0.01, ^∗∗∗^*P* < 0.001, ^∗∗∗∗^*P* < 0.0001; ns: *P* > 0.05.

6–8 weeks female NIH mice (Charles River Laboratory) were randomly divided into different groups and housed in the Animal Center at the School of Public Health (Sichuan University, Chengdu, China) under specific pathogen-free conditions at room temperature (24 ± 2 °C) and constant humidity (55% ± 10%) on a 12 h–12 h light–dark cycle. Regarding immunization, the groups of mice (*n* = 6) first received two doses of the mRNA vaccine (0.1 μg or 1 μg per mouse) intramuscularly,^16^ followed by two doses of the RBD-HR protein vaccine^17^ (10 μg per mouse) administered intranasally with a 21-day interval between each dose (Fig. 1A). Mice were intramuscularly immunized with two doses of phosphate buffer saline solution (PBS), a low dose (0.1 μg per mouse) of the mRNA vaccine, or a high dose (1 μg per mouse) of the mRNA vaccine, which acted as the control groups. The volume of intramuscular immunization was 100 μL and that of intranasal immunization was 20 μL.

### Sample collection and single-cell suspension preparation

Fourteen days after the last immunization, samples were collected for the subsequent experiments as previously described.^10^^,^^17^^,^^18^ Briefly, blood samples were collected from the ocular veniplex under light anesthesia with isoflurane inhalation. The sera were collected from the upper layer after centrifugation at 1000 *g* for 10 min twice, inactivated by heating at 56 °C for 0.5 h, and preserved at -80 °C for the ensuing experiments. After exposing the trachea, a blunt needle was inserted into the exposed trachea and secured in place. The lung or nasal cavity was rinsed three times with 1 mL or 0.6 mL PBS including 0.05% bovine serum albumin/BSA (0.05% BSA-PBS, 821005, Sigma–Aldrich, USA) through the trachea, respectively. The supernatant of nasal lavage fluid (NLF) and BALF was collected after centrifugation at 300 *g* for 5 min and preserved at -80 °C prior to use. The cell pellets of BALF were collected for flow cytometry analysis. Lung tissues were dissected into small pieces and digested with 1% collagenase type I (17018029, Thermo Fisher Scientific, USA) and 0.05% collagenase type IV (17104019, Thermo Fisher Scientific, USA) at 37 °C in a shaking incubator. Mediastinal lymph node, inguinal lymph node, and spleens were sampled by immersing them in 0.05% BSA-PBS and subsequently gently grounding them through 70 μm white nylon cell strainers (352350, BD Biosciences, USA) to finally acquire single cell suspensions. If the single cell suspension contained erythrocytes, it was further lysed for 5–8 min using an erythrocyte lysis buffer (R1010-500, Solarbio, China).

### ELISA

ELISA was employed to determine RBD-HR-specific antibody responses in the serum, NLF, and BALF, as described in the previous study.^19^ Purified antigens RBD-HR were coated onto 96-well plates at 0.1 μg/well in a carbonate coating buffer at 4 °C overnight. Afterward, the coated plates were washed with PBST (PBS containing 0.05% Tween-20) to remove loosely adherent RBD-HR protein and blocked with PBST containing 1% BSA at 37 °C for 2 h. After washing, the sera were subjected to a serial dilution of 2-fold and then incubated in the RBD-HR-coated wells at 37 °C for 1.5 h. The plates were subsequently washed 3 times and incubated with 1:10,000 diluted HRP-conjugated goat anti-mouse IgG (H + L) secondary antibody (31430, Thermo Fisher Scientific, USA), followed by detection with TMB ELISA substrate (34029, Thermo Fisher Scientific, USA). After reacting at 25 °C for 10 min, a stop solution for TMB substrate (P0215, Beyotime, China) was added to terminate the reaction, and the plates were observed at 450 nm and 630 nm wavelengths within 0.5 h using a microplate reader. Regarding IgG subtypes and IgA detection, goat anti-mouse IgG1, IgG2a, IgG2b, IgG2c, IgG3, and IgA secondary antibodies (5300-05, Southern Biotech, USA; ab97255, Abcam, UK) were used at a dilution of 1:10,000. Endpoint titers were determined by the highest dilution at which the optical density values were higher than those of control samples.

### Inhibition of RBD binding to 293T/hACE2 cells

The inhibition of specific antibodies on RBD-Fc binding to hACE2-expressing cells was observed by flow cytometry as previously reported.^20^^,^^21^ Prototype and mutant recombinant SARS-CoV-2 RBD-Fc, including WT, B.1.617.2, and BA.1 (Sino Biological, China; Acro, China), were preincubated with 1:90 diluted sera at 37 °C for 0.5 h in 96-well plates. Then, the RBD-Fc/sera mixture was transferred into flow tubes and co-incubated with 293T/hACE2 cells (2.5 × 10^4^ per well) at 25 °C for 0.5 h. After removing the unbound RBD-Fc, PE anti-human IgG Fc antibody (410708, Biolegend, USA) was added, and the mixture was incubated at 4 °C for 0.5 h. Thereafter, the cells were washed with 0.05% BSA-PBS, and the cell pellet was resuspended in 0.05% BSA-PBS. Mean fluorescent intensity was quantified using a flow cytometer (ACEA Biosciences, USA), and the data were processed by NovoExpress software and GraphPad Prism 9.3.

### Pseudovirus neutralization

The neutralizing activity of immune sera to SARS-CoV-2 eGFP/luciferase-expressing pseudotyped viruses, including WT, B.1.617.2, BA.1, BA.2, BA.3, and BA.4/5 (Genomeditech, China), was visualized under a fluorescence microscope (Olympus, Japan), and then quantified by flow cytometry and a microplate reader (PerkinElmer, USA). After being inactivated at 56 °C for 0.5 h, the serum samples were serially diluted from 10 to 21,870, and co-incubated with various pseudoviruses at 37 °C for 1 h. Next, 293T/hACE2 cells (1.2 × 10^4^ per well) were seeded into a 96-well plate and co-incubated with pseudoviruses at 37 °C for 48 h. The blockade of eGFP/luciferase-expressing pseudotyped virus (WT) and 293 T/hACE2 cells was observed by fluorescence microscopy and flow cytometry. Concurrently, the supernatants were extracted from the 96-well plates, and 100 μL fluorescein substrate reagent (RG056M, Beyotime, China) was added, and the mixture was allowed to react for 5 min. The firefly luciferase activity in the cells was detected by a microplate reader, and 50% pseudovirus neutralization titer (pVNT50) was calculated via nonlinear regression in GraphPad Prism 9.3.^17^

### Flow cytometry

Mononuclear cells obtained from BALF and tissues were transferred into flow tubes and stained with fluorescent-dye conjugated antibodies at 4 °C for 0.5 h. They were then stained with LIVE/DEAD™, PerCP/Cyanine5.5 anti-mouse CD45 antibody, FITC anti-mouse/human CD11b, PE/Cyanine7 anti-mouse CD11c antibody, APC anti-mouse CD103 antibody, Brilliant Violet 711™ anti-mouse I-A/I-E antibody, and PE anti-mouse CD86 antibody to identify CD103^+^ dendritic cells (DCs) and activated CD103^+^ DCs. The cells were also stained with PerCP/Cyanine5.5 anti-mouse CD3 antibody, Brilliant Violet 421™ anti-mouse CD4 antibody, APC anti-mouse CD8a antibody, PE anti-mouse/human CD44 antibody, FITC anti-mouse CD69 antibody, and Brilliant Violet 711™ anti-mouse CD103 antibody to identify T cells, memory T cells, and T_RM_. Additionally, the cells were stained with PerCP/Cyanine5.5 anti-mouse CD3 antibody, PE anti-mouse CD19 antibody, APC anti-mouse CD4 antibody, FITC anti-mouse CD185 (CXCR5) antibody, and Brilliant Violet 421™ anti-mouse CD279 (PD-1) antibody to visualize T follicular helper (Tfh) cells. Finally, germinal center (GC) B cells were identified by staining with PerCP/Cyanine5.5 anti-mouse CD3 antibody, PE/Cyanine7 anti-mouse/human CD45R/B220 antibody, APC anti-MU/HU GL7 antigen (T/B Cell Act. Marker) antibody, and FITC anti-mouse CD95 (Fas) antibody. All the immune cells were identified using previously established gating schemes,^10^^,^^19^ and the antibodies for flow cytometry were acquired from BioLegend.

To investigate SARS-CoV-2-specific cytokine secretion, cell suspensions from lung tissue were collected and re-stimulated with the SARS-CoV-2 peptide pool (DD9106, Vazyme, China). In short, a single-cell suspension (1.5 × 10^6^ per well) was added into 12-well plates and stimulated with a peptide pool (1 μg/well) at 37 °C for 12 h. 4–6 h before cell sample collection, cell secretion was blocked with Brefeldin A (420601, BioLegend, USA). The cells were thereupon incubated with PerCP/Cyanine5.5 anti-mouse CD3 antibody, APC anti-mouse CD4 antibody, FITC anti-mouse CD8a antibody, and PE anti-mouse/human CD44 antibody for labeling the plasma membrane of T cells. Concerning intracellular cytokine staining, the cells were fixed and permeabilized with a Fixation/Permeabilization Kit (BD Biosciences, USA) and incubated with PE/Cyanine7 anti-mouse interferon-γ antibody, Brilliant Violet 510™ anti-mouse TNF (tumor necrosis factor)-α antibody, and Brilliant Violet 711™ anti-mouse IL (interleukin)-2 antibody at 25 °C for 1 h. Following staining with DAPI (4′,6-diamidino-2-phenylindole; C1005, Beyotime, China) at 25 °C for 3 min, the cells were washed with a buffer solution and resuspended in PBS for flow cytometry analysis.

### Statistical analysis

All statistical analyses were performed using GraphPad Prism 9.3. The number of experimental animals and comparisons between groups are detailed in the figure legends. Results were expressed as mean ± standard error of mean (SEM) and *P* values were calculated by one-way ANOVA (*n* = 6, ^∗^*P* < 0.05, ^∗^^∗^*P* < 0.01, ^∗^^∗^^∗^*P* < 0.001, ^∗^^∗^^∗^^∗^*P* < 0.0001; ns: *P* > 0.05).

## Results

### Intranasal boosting with the RBD-HR vaccine elicited a robust humoral response

Mice were intramuscularly immunized with an mRNA vaccine (0.1 μg or 1 μg per mouse) encoding Delta full-length SARS-CoV-2 S protein,^16^ followed by intranasal immunization with 10 μg RBD-HR vaccine containing Delta-derived self-assembled trimeric protein^17^ (Fig. 1A). Serum, NLF, and BALF were collected 14 days after the last immunization to analyze the level of RBD-HR-specific antibodies. Compared with the mRNA vaccine alone, sera from mice intranasally boosted with two doses of RBD-HR vaccine exhibited a higher level of anti-RBD-HR IgG. The geometric mean titer of the 0.1 mRNA, 0.1 mRNA + RBD-HR, 1 mRNA, and 1 mRNA + RBD-HR groups attained 2.6 × 10^4^, 3.7 × 10^5^, 1.5 × 10^6^, and 2.7 × 10^6^, respectively, as evidenced by a 14.2-fold increase in the low-dose heterologous group and a 1.8-fold increase in the high-dose heterologous group (Fig. 1B, G). The intranasal booster also induced higher levels of RBD-HR-specific IgG subclass antibodies, including IgG1, IgG2a, IgG2b, IgG2c, and IgG3, whilst the titers of IgG1 were markedly higher than those of IgG2a and other subclasses (Fig. 1C, H). The analysis of Th1/Th2 antibody response determined that heterologous intranasal boost with the RBD-HR vaccine elicited a mixed Th1/Th2 immune response with a predominant Th2-skewing capability, which was consistent with the observations of previous studies.^22^^,^^23^ Moreover, heterologous boosting with the RBD-HR vaccine was demonstrated to be more effective in eliciting specific serum IgA in mice (Fig. 1D, I). However, intramuscular immunization with the mRNA vaccine was insufficient to induce a strong mucosal antibody response, whereas intranasal booster could effectively promote SIgA production in NLF and BALF (Fig. 1E, F, J, K). Thus, intramuscular administration of two doses of mRNA vaccine exhibited suboptimal immunogenicity and intranasal boosting with RBD-HR vaccine was able to induce potent mucosal and systemic antibody responses.

### Intranasal boosting with RBD-HR vaccine inhibited the binding of RBD to hACE2

**Figure 2:**
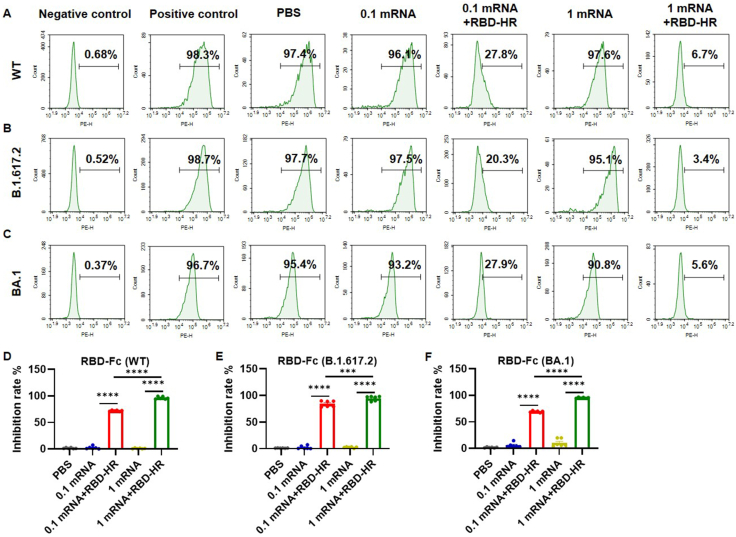
Immune sera inhibited the binding of RBD to hACE2. Fourteen days after the last immunization, the mouse sera were diluted to 1:90. **(A–C)** Representative graphs of flow cytometry displaying the inhibitory effect of immune sera against different types of RBD binding to ACE2 receptors, including WT RBD (A), B.1.617.2 RBD (B), and BA.1 RBD (C). **(D–F)** The inhibitory rate of immune sera against different types of RBD binding to hACE2 receptors, including WT RBD (D), B.1.617.2 RBD (E), and BA.1 RBD (F). Results were presented as mean ± SEM, and *P* values were determined by one-way ANOVA. *n* = 6, ^∗^*P* < 0.05, ^∗∗^*P* < 0.01, ^∗∗∗^*P* < 0.001, ^∗∗∗∗^*P* < 0.0001; ns: *P* > 0.05.

To investigate the inhibitory efficiency of immune sera, RBD-Fc fusion proteins (WT, B.1.617.2, and BA.1) were involved in the RBD-hACE2 binding assay. Flow cytometric analysis determined that the immune sera from the mRNA vaccinated group exhibited a poor blocking ability of RBD (WT) binding to 293T/hACE2 cells, which was consistent with the high positive rate of RBD binding observed in the PBS group (>90%). Nevertheless, the binding of RBD (WT) to hACE2 was suppressed after incubating with the sera from heterologous intranasal boost groups at a dilution of 1:90 (Fig. 2A). The mean inhibitory rates of the PBS, 0.1 mRNA, 0.1 mRNA + RBD-HR, 1 mRNA, and 1 mRNA + RBD-HR groups were 1.32% ± 0.88%, 2.19% ± 2.39%, 71.96% ± 1.00%, 0.75% ± 0.64%, and 96.22% ± 2.17%, respectively, and the difference in inhibitory rates between the mRNA and mRNA + RBD-HR groups remained significant (Fig. 2D). Notably, immune sera from heterologous intranasal boost groups remarkably blocked RBD (B.1.617.2)- and RBD (BA.1)-hACE2 interactions from over 90% to less than 30% at a dilution of 1:90 (Fig. 2B, C), and exhibited a higher inhibitory rate compared with the mRNA groups (Fig. 2E, F). Altogether, the findings indicated that intranasal boosting with the RBD-HR vaccine may confer potential protectivity against prototype and mutated SARS-CoV-2.

### Broad-spectrum neutralizing activity

**Figure 3:**
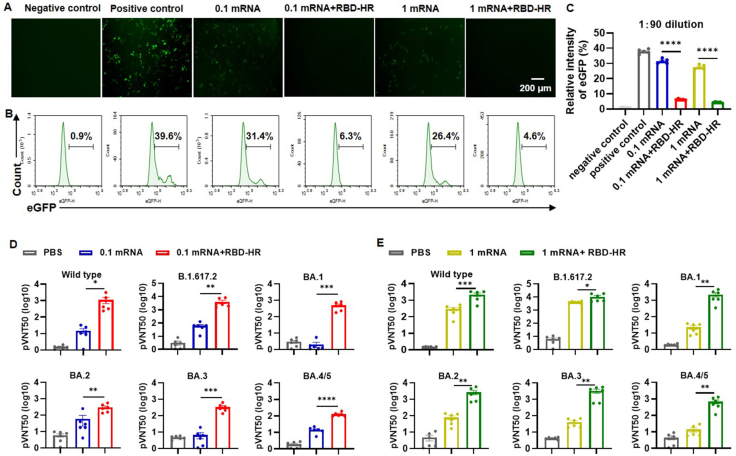
Immune sera inhibited the binding of SARS-CoV-2 pseudoviruses to hACE2. **(A–C)** Fourteen days after the last immunization, the mouse sera were diluted to 1:90. (A) Representative graphs of fluorescence microscopy illustrating the neutralization of immune sera to WT SARS-CoV-2 pseudovirus. Scale bar = 200 μm. (B) Representative graphs of flow cytometry depicting the neutralization of immune sera to WT SARS-CoV-2 pseudovirus. (C) Neutralizing activities of immune sera to WT SARS-CoV-2 pseudovirus characterized by the relative intensity of eGFP. **(D–E)** pVNT50 of immune sera against SARS-CoV-2 pseudoviruses, including WT, B.1.617, BA.1, BA.2, BA.3, and BA.4/5. Results were expressed as mean ± SEM, and *P* values were calculated by one-way ANOVA. *n* = 6, ^∗^*P* < 0.05, ^∗∗^*P* < 0.01, ^∗∗∗^*P* < 0.001, ^∗∗∗∗^*P* < 0.0001; ns: *P* > 0.05.

Neutralizing activity was investigated 14 days after the last immunization to assess the functional quality of the specific antibodies elicited by intranasal boosting with the RBD-HR vaccine. Significant increases in neutralizing antibodies against parental SARS-CoV-2 and variant pseudoviruses were observed after intranasal boosting with the RBD-HR vaccine in both low- and high-dose heterologous groups (Fig. 3). eGFP expression was significantly decreased in infected cells when prototype pseudovirus was incubated with the sera from heterologous intranasal boost groups at 1:90 dilution (Fig. 3A). Similarly, flow cytometric analysis was conducted to confirm the neutralizing effect of immune sera against pseudoviruses. The results demonstrated that the immune sera from heterologous groups were able to effectively neutralize prototype pseudovirus (Fig. 3B, C). Furthermore, intranasal boosting with the RBD-HR vaccine could effectively induce cross-neutralizing antibody responses against ancestral SARS-CoV-2 and major variants. In comparison to the low-dose mRNA groups, intranasal boosting with the RBD-HR vaccine led to a remarkable elevation in geometric mean titers of 50% neutralization against wild-type, B.1.617.2, BA.1, BA.2, BA.3, and BA.4/5 variants. The geometric mean titers of 50% neutralization for these variants were increased by 73.1-, 65.8-, 265.6-, 5.0-, 50.1-, and 8.8-fold, respectively (Fig. 3D). The geometric mean titers of 50% neutralization in the high-dose mRNA plus intranasal boosting vaccination group against wide-type and mutated pseudoviruses were increased by 7.4-, 2.5-, 99.0, 34.1-, 78.3-, and 49.6-fold, respectively, compared with the high-dose mRNA groups (Fig. 3E). The validation of neutralizing activity against pseudoviruses further corroborated the potential efficacy of the intranasally heterologous strategy.

### Respiratory mucosal T cell responses

**Figure 4:**
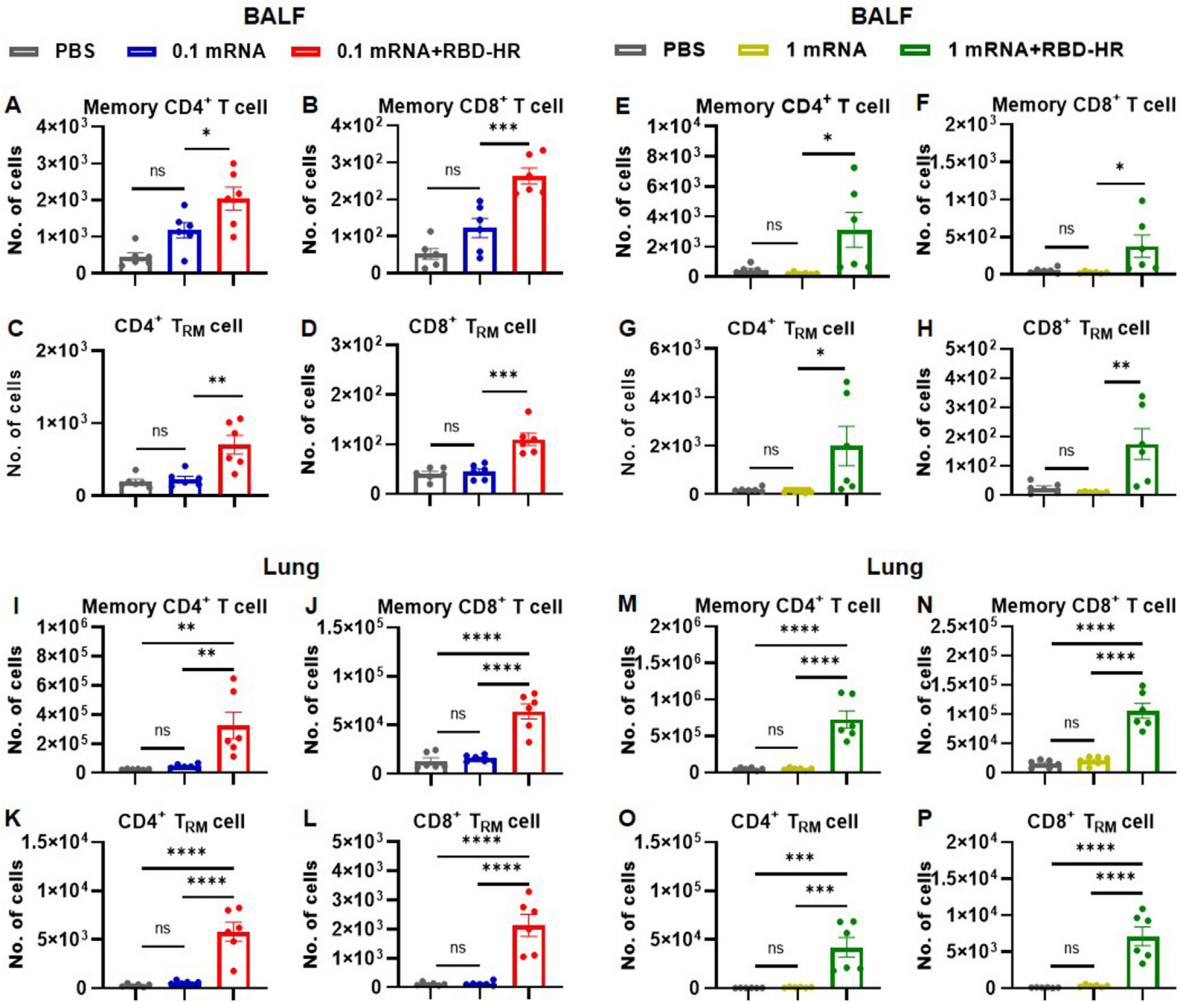
Intranasal boosting with the RBD-HR protein vaccine triggered memory T cell and T_RM_ responses. **(A–D)** Administration of low doses of the mRNA vaccine followed by the RBD-HR vaccine triggered memory T cell and T_RM_ responses in the BALF. **(E–H)** High doses of the mRNA vaccine, followed by the RBD-HR vaccine, triggered memory T cell and T_RM_ responses in the BALF. **(I–L)** Low doses of the mRNA vaccine, followed by the RBD-HR vaccine, triggered memory T cell and T_RM_ responses in the lungs. **(M–P)** High doses of the mRNA vaccine, followed by the RBD-HR vaccine, triggered memory T cell and T_RM_ responses in the lungs. Results were expressed as mean ± SEM, and *P* values were calculated by one-way ANOVA. *n* = 6; ^∗^*P* < 0.05, ^∗∗^*P* < 0.01, ^∗∗∗^*P* < 0.001, ^∗∗∗∗^*P* < 0.0001; ns: *P* > 0.05.

**Figure S1:**
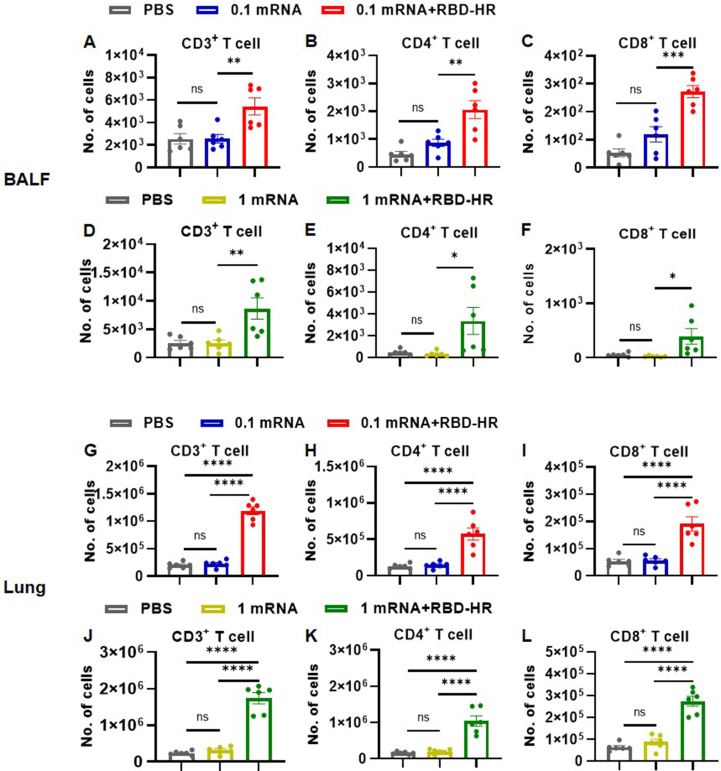
Sequentially intranasal immunization with the RBD-HR vaccine triggered T cell response. **(A–F)** Administration of the mRNA vaccine followed by the RBD-HR vaccine elicited T cell response in the BALF. **(G–L)** Administration of the mRNA vaccine followed by the RBD-HR vaccine elicited T cell response in the lungs. Results were expressed as mean ± SEM, and *P* values were calculated by one-way ANOVA. *n* = 6; ∗*P* < 0.05, ∗∗*P* < 0.01, ∗∗∗*P* < 0.001, ∗∗∗∗*P <* 0.0001; ns: *P* > 0.05.

To investigate the impact of intranasal boosting with the RBD-HR vaccine on eliciting an effective cellular immune response in the respiratory tract, the number of T cells in BALF and lung tissue was observed. Our results showed that the groups that received two intranasal immunizations had a significant increase in the number of T cells (CD3^+^), helper T cells (CD3^+^ CD4^+^), and cytotoxic T cells (CD3^+^ CD8^+^) in BALF and lung tissue (Fig. S1). This finding suggested that intranasal boosting with the RBD-HR vaccine may stimulate the activation and differentiation of both CD4^+^ and CD8^+^ T cell subsets within the respiratory tract. Furthermore, compared with the mRNA vaccine groups, most memory CD4^+^ T cells (CD4^+^ CD44^+^) and memory CD8^+^ T cells (CD8^+^ CD44^+^) resided within BALF (Fig. 4A, B, E, F) and lung tissue (Fig. 4I, J, M, N) in the heterologous groups. It is worthwhile emphasizing that T_RM_ is regarded as a distinct subclass of memory T cells settling within non-lymphoid tissues, including mucosal surfaces and lung tissue, and can provide rapid and effective immune responses upon re-exposure to SARS-CoV-2.^24^ Furthermore, CD4^+^ T_RM_ can assist B cells in the production of neutralizing antibodies and activate multiple immune effectors such as CD8^+^ T cells, while CD8^+^ T_RM_ can directly kill infected cells and secrete anti-viral cytokines that can restrict the replication and spread of SARS-CoV-2.^25^ Intriguingly, a significant increase in the population of CD4^+^ T_RM_ (CD4^+^ CD44^+^ CD69^+^ CD103^+^) and CD8^+^ T_RM_ (CD8^+^ CD44^+^ CD69^+^ CD103^+^) was noted in BALF (Fig. 4C, D, G, H) after boosting with the RBD-HR vaccine, which was consistent with the findings in lung tissue (Fig. 4K, L, O, P). Overall, these findings demonstrated that intranasal boosting with the RBD-HR vaccine-induced protective immunity through the establishment of T_RM_ in the respiratory mucosa and lung parenchyma, highlighting that intranasal boosting with the RBD-HR vaccine may provide long-lasting protection against SARS-CoV-2.

### CD103^+^ DCs activation in the lungs

**Figure 5:**
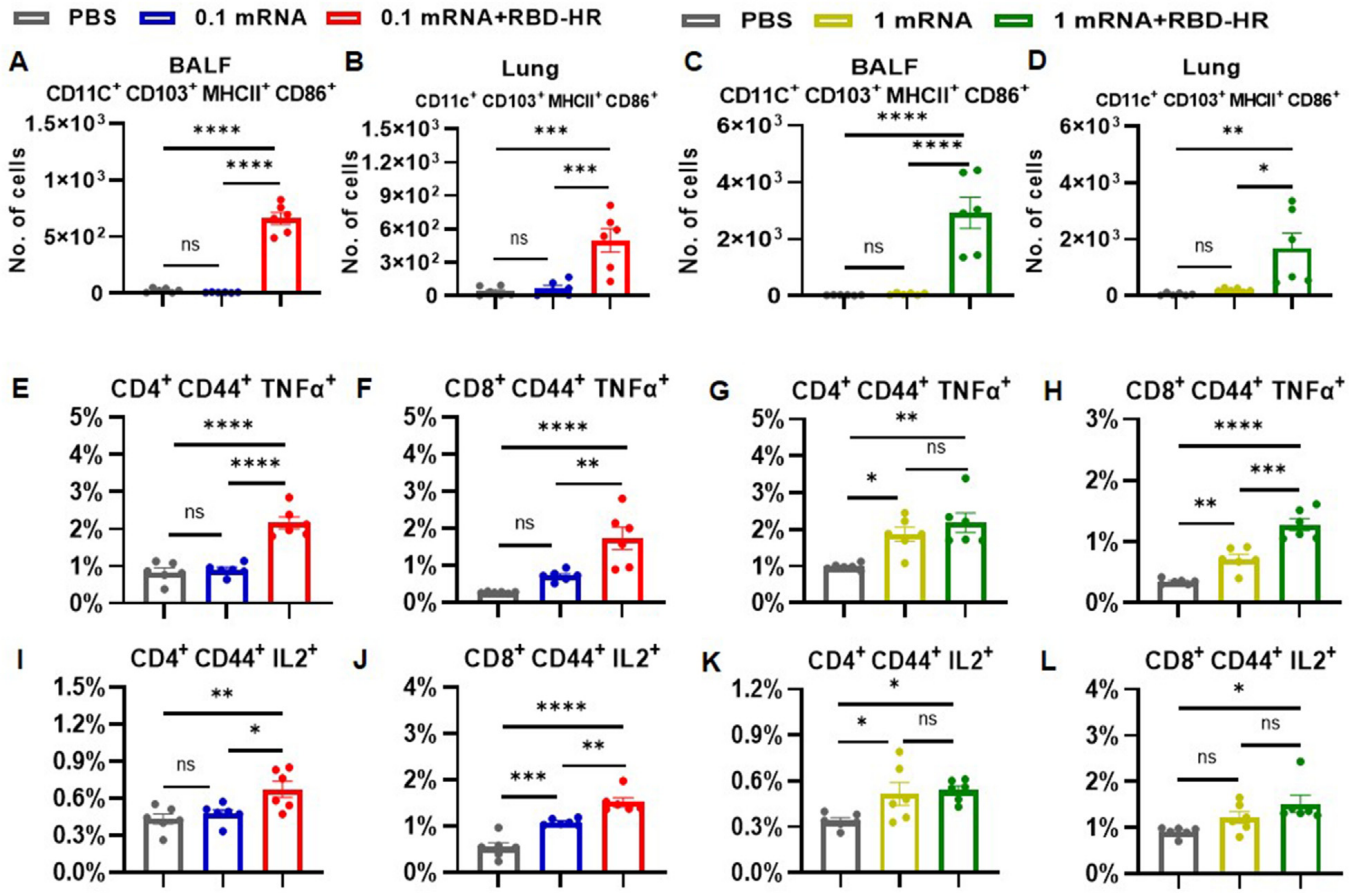
Intranasal boosting with the RBD-HR protein vaccine activated CD103^+^ DCs and triggered T cells to secret Th1 cytokines in the lungs. **(A–D)** Administration of the mRNA vaccine followed by the RBD-HR vaccine promoted DCs activation in BALF and lung. **(E–H)** The percentages of memory CD4^+^ or memory CD8^+^ T cells secreting TNF-α. **(I–L)** The percentages of memory CD4^+^ or memory CD8^+^ T cells secreting IL-2. Results were expressed as mean ± SEM, and *P* values were calculated by one-way ANOVA. *n* = 6; ^∗^*P* < 0.05, ^∗∗^*P* < 0.01, ^∗∗∗^*P* < 0.001, ^∗∗∗∗^*P* < 0.0001; ns: *P* > 0.05.

According to previous studies, pulmonary CD103^+^ DCs can be divided into two subsets: resident DCs that develops from local precursors in the lung parenchyma, and migratory DCs that originates from blood monocytes and migrates from the airways to the distal tissue.^26^ BALF CD103^+^ DCs are predominantly migratory DCs, whereas lung tissue CD103^+^ DCs also encompasses resident DCs.^27^ Activated CD103^+^ DCs, which express major histocompatibility complex class II and CD86 molecules, is essential for the generation and retention of T_RM_, making a crucial contribution to the initiation and maintenance of the immune response against pulmonary infections.^27^^,^^28^ Therefore, the activation of CD103^+^ DCs in the lungs was investigated after intranasal boosting with the RBD-HR vaccine. Intramuscular immunization with low- or high-dose mRNA vaccine could hardly promote the activation of CD103^+^ DCs in BALF and lung tissue consisting of the PBS groups (Fig. 5A–D). In contrast, intranasal boosting with the RBD-HR vaccine was conducive to the activation of CD103^+^ DCs in BALF, increasing the number of activated CD103^+^ DCs in the intranasal boost groups by up to 94.6- and 52.1-fold compared with the low- and high-dose mRNA groups, respectively (Fig. 5A, C). Similar results were also found in the lung tissue. More specifically, the proportion of activated CD103^+^ DCs was markedly higher in intranasal booster groups than that of the PBS and mRNA vaccine groups. Compared with the mRNA vaccine groups, the number of activated CD103^+^ DCs in the heterologous groups was increased by 7.4- and 8.8-fold, respectively (Fig. 5B, D). These findings authenticated that intranasal boosting with the RBD-HR vaccine significantly boosted CD103^+^ DCs activation.

### Th1-biased cellular immune response *ex vivo*

Our findings revealed that intranasal boosting with RBD-HR promoted mixed Th1/Th2 immune responses that were dominated by Th2 polarization. To further identify the Th1 response elicited by intranasal boosting with the RBD-HR vaccine, cells isolated from the lung tissue were incubated with a SARS-CoV-2 peptide pool *ex vivo*, and cytokine-secreting T cells were observed by intracellular cytokine staining. In contrast to intramuscular immunization with low-dose mRNA vaccine, which failed to effectively induce memory T cells to secrete TNF-α and IL-2 (Fig. 5E, F, I, J), intranasal boosting with the RBD-HR vaccine resulted in a substantial increase in the proportion of memory CD4^+^ T cells and memory CD8^+^ T cells secreting TNF-α and IL-2 (Fig. 5E, F, I, J). Moreover, cytokine production was partially facilitated by intramuscular injection of a high-dose of mRNA vaccine, while intranasal boosting with the RBD-HR vaccine further enhanced the secretion of TNF-α and IL-2 by memory T cells compared with the PBS control groups (Fig. 5G, H, K, L). Taken together, these results suggested that intranasal boosting with the RBD-HR vaccine induced a Th1-biased cellular immune response characterized by TNF-α and IL-2 secretion, which has significant implications in the prevention of infection and limiting the spread of SARS-CoV-2.

### Robust germinal center responses in lymph nodes and spleen

**Figure 6:**
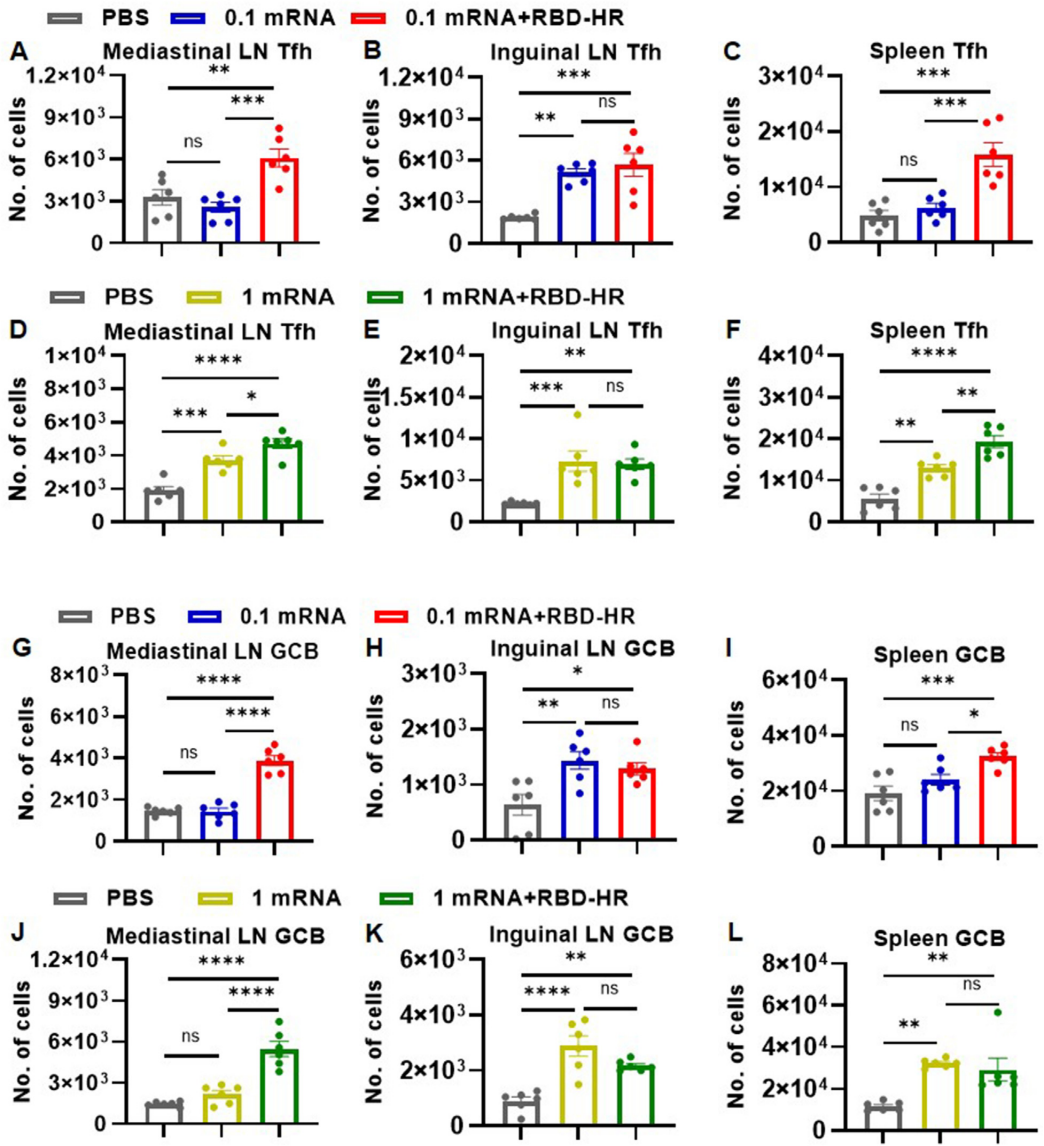
Intranasal boosting with the RBD-HR protein vaccine promoted GC B cell and Tfh cell generation. **(A–F)** Administration of the mRNA vaccine followed by the RBD-HR vaccine boosted the production of Tfh cells in the mediastinal lymph node (LN) (A, D), inguinal LN (B, E), and spleen (C, F). **(G–L)** Administration of the mRNA vaccine followed by the RBD-HR vaccine, boosted the production of GC B cells in mediastinal LN (G, J), inguinal LN (H, K), and spleen (I, L). Results were expressed as mean ± SEM, and *P* values were calculated by one-way ANOVA. *n* = 6; ^∗^*P* < 0.05, ^∗∗^*P* < 0.01, ^∗∗∗^*P* < 0.001, ^∗∗∗∗^*P* < 0.0001; ns: *P* > 0.05.

Both intramuscular and intranasal vaccinations lead to a significant activation and proliferation of immune cells within mediastinal lymph nodes, inguinal lymph nodes, and spleen.^29^^,^^30^ Tfh cells and GC B cells have been implicated as critical components in generating germinal centers for protective immune responses against SARS-CoV-2.^31^^,^^32^ To determine whether intranasal boosting with RBD-HR can induce a robust germinal center reaction, the production of Tfh cells and GC B cells was examined in these three major immune organs in mice. After intramuscular injection of 0.1 μg mRNA vaccine, the number of Tfh and GC B cells in inguinal lymph nodes was increased, but no significant difference was identified in mediastinal lymph nodes and spleen (Fig. 6A–C, G–I). A low dose of mRNA vaccine may not induce detectable levels of immune response in distal lymphoid tissue, which was in line with the results of previous studies.^33^^,^^34^ When the mice were administrated with a low dose of mRNA vaccine, the distribution of Tfh and GC B cells increased in the mediastinal lymph nodes, inguinal lymph nodes, and spleen (Fig. 6D–F, J–L). Notably, intranasal boosting with the RBD-HR vaccine maintained the generation of Tfh cells and GC B cells in the major immune organs and increased their distribution in the mediastinal lymph nodes and spleen (Fig. 6). Our comparative analysis exposed that intranasal boosting with the RBD-HR vaccine promoted Tfh and GC B cell generation, which could be a superior approach to inducing robust cellular immune responses and providing substantial protection against COVID-19 infection.

## Discussion

**Figure S2:**
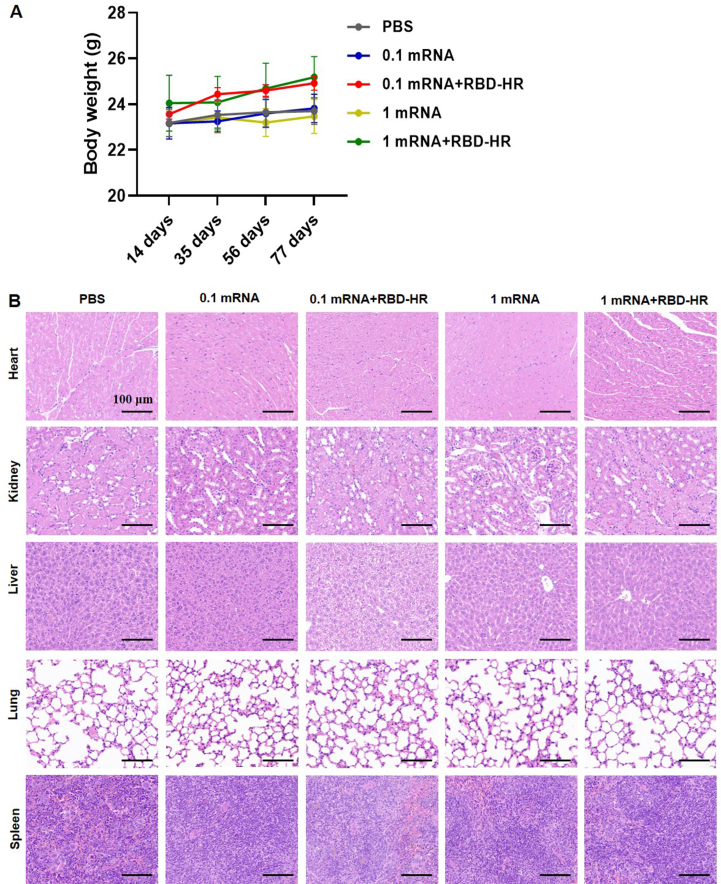
Safety investigation of the mRNA vaccine followed by RBD-HR vaccine. **(A)** Body weight of immunized mice. **(B)** The vital organs of mice (heart, kidney, liver, lung, spleen) were fixed and then stained with hematoxylin and eosin. Scale bars = 100 μm.

Following initial priming with intramuscular vaccination, intranasal booster vaccination is regarded as a promising strategy to evoke mucosal and systemic immunity against SARS-CoV-2. In this study, the immunogenicity and protective efficacy of intranasal boost immunization with the RBD-HR protein vaccine after systemic prime immunization with the mRNA vaccine were investigated. Intranasal boosting with the RBD-HR vaccine following mRNA vaccination led to significant increases in the levels of binding antibodies and neutralizing antibodies. The regimen also elicited extensive memory T cell responses, CD103^+^ DCs activation in respiratory mucosa, and enhanced Tfh and GC B cell generation in critical immune organs. Throughout the vaccination period, all mice remained in healthy condition, exhibiting stable weight changes and no significant pathological alterations in the histopathological examinations of different vital organs (Fig. S2).

A standard dose of mRNA vaccine is associated with an increased risk of adverse reactions, such as myocarditis, pulmonary embolism, stroke, thrombosis, *etc*.^35^ In contrast, a low dose of mRNA vaccine can limit the incidence of adverse reactions.^36^ Earlier studies have described that specific IgG binding antibodies could be detected in mice seven days after intramuscular injection of 0.5–20 μg mRNA vaccine, with the antibody persisting for at least 35 days.^37^ Meanwhile, two doses of one-quarter the standard dose (25 μg/dose) of the mRNA-1273 vaccine induced robust CD4^+^ helper T cells, CD8^+^ cytotoxic T cells, and humoral immune responses, which was comparable to natural infection.^38^ Herein, low-dose mRNA vaccine (0.1 μg or 1 μg per mouse) as prime vaccination initiated systemic immune responses, while a standard dose of the RBD-HR vaccine as boost vaccination enhanced both systemic and mucosal immune responses. Therefore, in situations where the supply of mRNA vaccine is limited, decreasing the dose of mRNA and adopting a heterologous intranasal boosting strategy can allow vaccine administration to a higher number of individuals while minimizing adverse reactions and providing extensive protective immunity.

The development of multivalent vaccines that target several various variants of the virus could prove to be crucial in achieving protection against constantly mutated SARS-CoV-2.^39^ Receptor-binding inhibition assay and pseudovirus neutralization assay are widely employed to evaluate the blockade or neutralizing capacity of immunized sera against viral infection, given that they mimic viral infection while limiting the risks associated with authentic viruses.^40^ Our findings showcased the potential benefits of mRNA vaccine plus intranasal boosting with RBD-HR for the generation of high titers of specific IgG in the serum, thereby effectively blocking or neutralizing prototype SARS-CoV-2 and variants (Figure 1, Figure 2, Figure 3). Notably, the SARS-CoV-2 vaccine shared the same mutations that can elicit a stronger neutralizing antibody response against the homologous variant.^41^ In the present study, the mRNA vaccine contained Delta full-length S protein sequence while the RBD-HR vaccine conferred L452R and T478K mutations. As anticipated, the mRNA vaccine plus the RBD-HR vaccine was highly effective in evoking neutralization against the binding of RBD (Delta) and pseudovirus (B.1.617.2) to hACE2 receptors. However, owing to the stronger immune evasion capacity of Omicron strains, the neutralizing abilities generated by boosting with the RBD-HR vaccine against the Omicron variants, including BA.1, BA.2, BA.3, and BA.4/5, were marginally lower, which was consistent with the observation of previous literature reports.^42^, ^43^, ^44^, ^45^ Further research is warranted to design broad-spectrum vaccines that can provide robust and long-lasting protection against circulating and emerging SARS-CoV-2 variants.

The next generation of immunization strategies should consider both the multivalency and route of administration of SARS-CoV-2 vaccines. Except for multivalent vaccines, the heterologous intranasal boost strategy has emerged as an effective preventive approach against SARS-CoV-2, offering protection by inducing robust immune responses in all three arms of the immune system, namely, humoral, cellular, and mucosal immunity. Humoral immunity is associated with the production of antigen-specific antibodies, while cellular immunity is mediated by immune cells and is crucial for recognizing and eliminating virus-infected cells. Mucosal immunity, on the other hand, provides defensive protection against SARS-CoV-2 that enters the body through the respiratory tract.^2^^,^^25^ Intriguingly, SIgA antibodies are the predominant immunoglobulin in mucosal immunity and serve as an indispensable component against SARS-CoV-2 at the mucosal surfaces of the respiratory tract.^46^ Notably, SIgA neutralizes SARS-CoV-2 by binding to RBD with high affinity before infecting epithelial cells and can also promote the clearance of the virus by recruiting immune cells to the site of infection.^47^^,^^48^ A prior study has identified that the presence of SIgA at mucosal surfaces can trigger neutrophils to undergo NETosis, resulting in the production of web-like structures that can trap and kill the virus to effectively limit the spread of SARS-CoV-2.^49^ Following heterologous intranasal immunization, the mRNA vaccine plus the RBD-HR vaccine successfully induced high levels of specific SIgA in the NLF and BALF (Fig. 1). Besides, intranasal boosting with the N-RBD^WT^ vaccine or N-RBD^Omicron^ vaccine elicited higher cross-variant neutralizing antibody titers and SIgA in BALF, in agreement with our findings.^50^ These results indicated that intranasal boosting with the RBD-HR vaccine protected SARS-CoV-2 in both the upper and lower respiratory tracts.

Recent studies have provided compelling evidence that memory T cells can rapidly enhance local and systemic protection and maintain long-term specific immune responses, while T_RM_ and CD103^+^ DCs play an instrumental role in eliciting mucosal immune responses against respiratory viral infections.^51^ At the same time, T_RM_ exists for a relatively short period in the lungs and thus may compromise responses to subsequent SARS-CoV-2 infection.^52^ The use of heterologous prime-boost regimens can enhance the longevity of T_RM_ in the lungs. Specifically, intranasal boost vaccination is a more effective approach than intramuscular boost vaccination for inducing long-lasting cellular immune responses within the respiratory mucosa.^10^^,^^15^ As expected, our findings demonstrated that intranasal boosting with the RBD-HR vaccine increased the number of both CD4^+^ T_RM_ and CD8^+^ T_RM_ in BALF and lung tissues (Fig. 4). This may be attributed to the fact that intranasal delivery of vaccines targets mucosa-associated lymphoid tissues, which are specialized immune structures located in the respiratory and gastrointestinal tracts, allowing for the establishment of a long-lasting and potent local immune response.^46^^,^^53^ Moreover, the results revealed a higher proportion of activated CD103^+^ DCs in BALF and lung tissue (Fig. 5). CD103^+^ DCs, residing near the airway epithelium, are particularly critical in mediating T cell immune response against pulmonary infections and known to be excellent at cross-priming CD8^+^ T cells to elicit antigen-specific cytotoxic responses against viruses.^27^ Moreover, CD103^+^ DCs can present viral antigens to CD8^+^ T cells for the induction of CD8^+^ T_RM_ through the production of TGF-β^54^ and facilitate the differentiation of CD4^+^ T_RM_ to CD8^+^ T_RM_ and B cells in turn.^55^ Taken together, heterologous intranasal vaccination offers a promising approach for inducing memory T cells, T_RM_, and CD103^+^ DCs, providing long-lasting protection against SARS-CoV-2-induced respiratory infection. Further research is necessitated to illustrate the mechanisms underlying the induction and maintenance of T_RM_ and CD103^+^ DCs in order to optimize the design of intranasal vaccines for clinical translation.

Our research does have some limitations. To begin, the current study only evaluated the short-term induction of mucosal and systemic immunity following intranasal boosting with the RBD-HR vaccine, and future research should focus on evaluating the long-term immune response of the heterologous intranasal boosting strategy. Secondly, the immunized mice were not challenged with lethal SARS-CoV-2, limiting the evaluation of the actual protective efficacy and viral clearance rate of the heterologous intranasal boost strategy. Thirdly, NIH mice as the model animal may not fully reflect the immunological efficacy of the mRNA vaccine plus RBD-HR vaccine. In-depth studies involving various animal models such as rats, guinea pigs, rabbits, and macaques are therefore required. Despite the limitations, these findings validated that intranasal boosting with the RBD-HR vaccine after priming with mRNA vaccine can elite robust mucosal, humoral, and cellular immune responses. Our study provides a feasible approach to improve the immunogenicity of primary immunization and emphasizes the need for a mucosal booster strategy in conjunction with current intramuscular SARS-CoV-2 vaccines.

## Author contributions

Xiawei Wei conceived and conceptualized the work and strategy. Guangwen Lu and Xiangrong Song prepared the RBD-HR and the mRNA vaccines, respectively. Li Chen, Wenyan Ren, and Hong Lei carried out the animal experiments with the assistance of Jiayu Wang, Haiying Que, Dandan Wan, Aqu Alu, Dandan Peng, Minyang Fu, Weiqi Hong, and Yuhe Huang. Li Chen drafted the manuscript with the help of all other co-authors.

## Conflict of interests

The authors have no conflicts of interest to declare.

## Funding

This study was funded by the National Science Foundation for Excellent Young Scholars of China (No. 32122052) and the National Natural Science Foundation Regional Innovation and Development of China (No. U19A2003).

## Data availability

All data supporting the findings of this study are available upon reasonable request from the corresponding author.
